# In vitro optoacoustic flow cytometry with light scattering referencing

**DOI:** 10.1038/s41598-021-81584-y

**Published:** 2021-01-26

**Authors:** Markus Seeger, Andre C. Stiel, Vasilis Ntziachristos

**Affiliations:** 1 Chair of Biological Imaging (CBI) and Center for Translational Cancer Research (TranslaTUM), School of Medicine, Technical University of Munich, Munich, Germany; 2 Institute of Biological and Medical Imaging (IBMI), Helmholtz Zentrum München, Neuherberg, Germany

**Keywords:** Imaging and sensing, Photoacoustics, Flow cytometry, Reporter genes, High-throughput screening

## Abstract

Morphological and functional optoacoustic imaging is enhanced by dedicated transgene reporters, in analogy to fluorescence methods. The development of optoacoustic reporters using protein engineering and directed evolution would be accelerated by high-throughput in-flow screening for intracellular, genetically encoded, optoacoustic contrast. However, accurate characterization of such contrast is impeded because the optoacoustic signals depend on the cell’s size and position in the flow chamber. We report herein an optoacoustic flow cytometer (OA-FCM) capable of precise measurement of intracellular optoacoustic signals of genetically-encoded chromoproteins in flow. The novel system records light-scattering as a reference for the detected optoacoustic signals in order to account for cell size and position, as well as excitation light flux in the focal volume, which we use to reference the detected optoacoustic signals to enhance the system’s precision. The OA-FCM was calibrated using micrometer-sized particles to showcase the ability to assess in-flow objects in the size range of single-cells. We demonstrate the capabilities of our OA-FCM to identify sub-populations in a mixture of two *E. coli* stocks expressing different reporter-proteins with a precision of over 90%. High-throughput screening of optoacoustic labels could pave the way for identifying genetically encoded optoacoustic reporters by transferring working concepts of the fluorescence field such as directed evolution and activated cell sorting.

## Introduction

Optoacoustic (OA, also termed photoacoustic) imaging combines optical excitation with ultrasound detection to afford deep tissue imaging of endogenous contrast from hemoglobin, lipids, melanin, and other strong absorbers. This intrinsic contrast is often insufficient to study tissue morphology and function, including metabolic and neuronal processes, which has driven research into transgene OA labels^1–4^. However, current methods of developing and screening new transgene OA labels are inefficient because they are based on measuring OA signals from *E. coli* colonies on growth plates^5,6^, which limits throughput and the use of mammalian cells for library expression.

Optoacoustic flow cytometry (OA-FCM) could allow for high-throughput screening of variants of transgene OA labels expressed in bacterial or mammalian cells, enabling the use of directed-evolution strategies for rapid label development, analogous to the methods used to design fluorescent labels^7,8^. However, measuring and accurately quantifying intracellular OA contrast in-flow is challenging because (1) ultrasound transducers capable of detecting micrometer sized objects typically exhibit highly spatially-dependent signal gathering and (2) delivering light into a microfluidic flow chamber yields an inhomogeneous fluence distribution. Hence, the recorded OA signals are strongly affected by the cell’s size and position in the interrogation area, which impedes the direct assessment and comparison of the investigated contrast agents.

Previously introduced OA-FCMs attempted to correct in-flow OA signals of cells by employing a second modality to infer a cell’s size and position. For example, fluorescence signals from cellular labels have been used to characterize the uptake of nanoparticles (e.g. gold nanorods, graphene) in single cells and enabled the assessment of the cell–particle interaction and the particle’s toxicity^9–11^. Ultrasound-backscattering from individual cells has been used for the label-free determination of cell size, which enabled the OA-based discrimination and dual-modal enumeration of red and white blood cells^12^. However, fluorescence signals are not independent of OA signals because they either originate from the identical optical absorption or require additional fluorescent labeling of the cells, both adding complexity and ambiguity to OA signal correction. Ultrasound-backscatter is independent of OA signals, but typically has poorer spatial resolution, which limits the achieved accuracy when implemented to normalize OA signals. Thus, there is a need for OA-FCMs to implement a method that is both independent of the OA modality and precise enough to enable accurate high-throughput assessment of the cell size and position; allowing the screening of variant libraries of potential transgene labels with OA-tailored photophysics.

Here we explore light scattering (LS) as a different type of normalization, which is independent from OA detection and cell absorptions. We hypothesized that recorded LS data would enable accurate correction of OA signals measured in-flow. Reading side scatter (90°) to measure the cells internal granularity, and forward scatter (0°) to measure cell size and surface area, has been shown to be an appropriate strategy to correct in-flow fluorescence data and afford higher accuracy readings^13^. Furthermore, LS scales with light fluence and, thus, the local intensity of the optical focal volume. This information would enable OA signals to be referenced to the actual optical excitation acting on the cells to afford position-independent and comparable signals. For that, *E. coli* cells can be considered as rod-shaped bacteria of ~ 4 µm length and ~ 1 µm diameter whose scattering can be approximated with Mie theory using the T-matrix method for a prolate spheroid^14–17^. Considering further that hydrodynamic forcers orient the long axis of spheroidal objects with the Poiseuille flow profile, the scattering intensity at an angle $\uptheta$ can be described as ${\mathrm{I}}_{\mathrm{s}}\left(\uptheta \right)=2\uppi {\mathrm{S}}_{11}(\uptheta )\bullet {\mathrm{I}}_{\mathrm{i}}$ based on the spheroid-specific Mueller matrix element ${\mathrm{S}}_{11}$^14–17^. Hence, the recorded scattering intensity at a given angle for approximately identical particles or cells is linearly proportional to the local light intensity.

We present a novel OA-FCM prototype that incorporates LS in order to infer the cell’s size and position in the focal volume and excitation light intensity. We reasoned that referencing intracellular OA signals to cellular LS would allow precise characterization and distinguishing of OA contrast agents in single-cells. We selected detection at 45°, as scattering at this angle primarily assesses the cells’ sizes and experienced fluence while being only marginally affected by intracellular absorption that would decrease the scattering intensity when recording at 0°. Furthermore, orienting the LS detector in the same direction as the flow ensures sensing of all cells passing through the optical focal volume, which would not be guaranteed by an orientation perpendicular to the flow. This allows precise in-flow measurement of OA signals, accurately normalized to the cell’s properties and its experienced optical excitation. Using micrometer sized particles and beads, we demonstrate the system’s ability to reference OA readings to LS signals in-flow, and in the size range of single cells. The referencing of OA contrast further enables the differentiation of transgene reporters expressed in cells, which only differ in their OA contrast by a factor of < 2. The sensitivity of our LS-referenced OA-FCM towards intracellular OA contrast could enable high-throughput screening of transgene OA reporters.

## Results

### The optoacoustic flow cytometer

**Figure 1:**
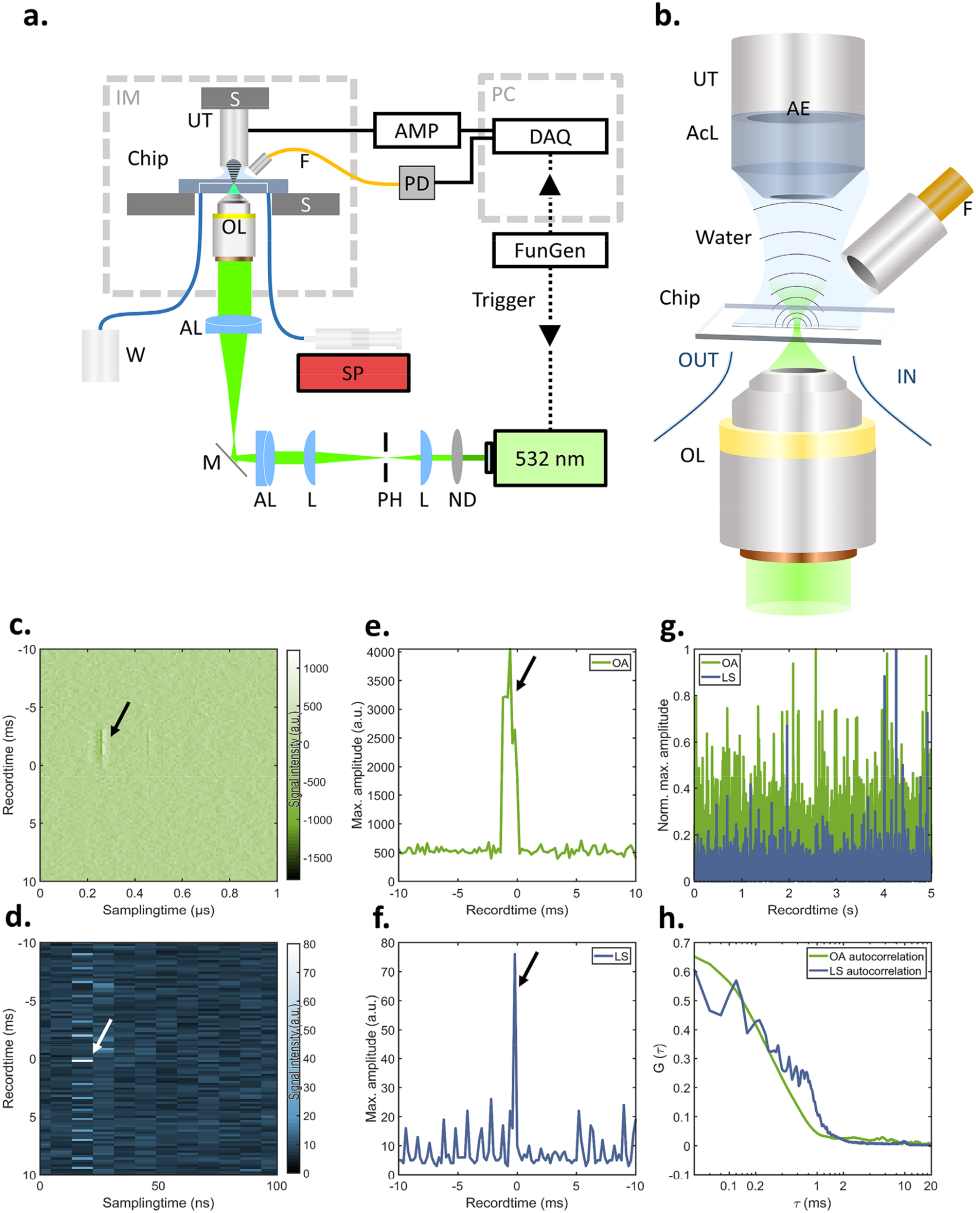
Schematic depiction and validation of the OA-FCM system. (**a**) The OA-FCM system is based on focusing a 532 nm laser into a microfluidic channel, which is connected to a syringe pump for controlling the flow of suspensions of particles or cells. OA and LS signals are recording simultaneously. (**b**) Close-up depiction of the microfluidic interrogation area. Raw (**c**) OA and (**d**) LS signal detected from a single bead indicated by an arrow flowing through the interrogation area flowing at 100 μL/min. (**e**) OA and (**f**) LS maximum amplitude projection of the record window shown in (c) and (d). (**g**) OA- and LS-trajectories recorded over 5 s show transits of individual beads. (**h**) Auto-correlation curves of the OA and LS trajectories in (e) reveal interrogation time of ~ 300 μs. Abbreviations: AE: active element; AcL: acoustic lens; AL: achromatic doublet lens; AMP: low noise amplifier; AWG: arbitrary waveform function generator; DAQ: data acquisition card; IM: inverted microscope; L: planoconvex lens; M: dielectric mirror; ND: neutral density filter; OL: microscope objective lens; PH: pinhole; S: high-precision motorized stage; SP: syringe pump; UT: Ultrasound transducer; W: Waste.

The designed OA-FCM (Fig. 1a,b, methods section, and described in the preprint of this manuscript^18^) consists of a 50 kHz pulsed 532 nm laser excitation focused into a standard 1000 μm × 100 μm microfluidic channel, through which the cells are guided. The resulting ultrasound signals are recorded by a 50 MHz focused transducer in perpendicular orientation, while LS signals are detected by a fiber-coupled photodiode in an orientation 45° angled to the OA detection.

To test the system’s capabilities of sensing OA and LS signals of cell-sized entities, we first measured a black 10 µm polystyrene bead as a reference specimen. Figure 1c,d depicts the transit of a single polystyrene beads sensed by OA and LS, respectively. The OA signal from the bead follows in first approximation a Gaussian curve, as expected, whereas the LS signal presents a sharp peak occurring at the end of the OA curve. The time lag between the two signals might be due to the LS detection being tilted and pointing in the same direction as the microfluidic flow, which causes LS signals to be detected at the end of an object’s passage. Figure 1e–f show the corresponding frequency filtered and Hilbert transformed maximum amplitude trajectories. These measurements suggest that the proposed OA-FCM system enables the measurement of transits for particles that are approximately 10 µm in diameter. Figure 1g plots the trajectories of simultaneously recorded OA and LS signals over a recorded time of 5 s, capturing single transit events above background noise. Over 90% of the events appear in both OA and LS signal recordings. As depicted in Fig. 1h, both autocorrelations of the OA and LS trajectories exhibit a correlation time of ~ 300 μs. Theoretically, a flow rate of 100 μL/min through a channel with a cross-section of 1000 µm × 100 μm leads to an average flow speed of 16.6 mm/s and a Reynolds number of ~ 3.03. Measuring beads of 10 μm diameter at the center of the microfluidic chip, at which the flow speed is expected to be double the average flow speed based on a laminar Poiseuille flow profile, this should result in an interrogation time of ~ 300 μs, i.e. ~ 15 pulses per bead at a repetition rate of 50 kHz and assuming an arbitrary small optical focus. We found a correlation time of ~ 280 µs (Fig. 1h), which confirms proper alignment of the system and accurate control of the flow speed.

### OA-FCM calibration

We characterized the system using polystyrene beads (µB; nominal diameter: 10 µm), carbon nanoparticles (CNP; size distribution: 2–12 µm), and *E. coli* cells expressing chromoprotein labels in order to estimate its performance with specimens of varying size and signal characteristics, as described in the preprint of this manuscript^18^.

**Figure 2:**
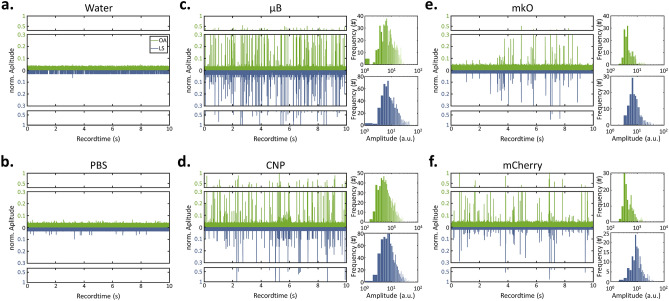
Characterization of in-flow signals at 100 μL/min. Normalized OA and LS trajectories of (**a**) water as the solvent for μB and CNPs as well as of (**b**) PBS as the buffer for the cell suspension exhibit no detected transit event over 10 s record time. Water-suspended (**c**) μB and (**d**) reveal OA and LS signals of transits of individual particles. Unnormalized histograms are shown Gaussian-shaped distribution over the entire 1 min measurement. Normalized OA-FCM trajectories of *E. coli* expressing (**e**) mKO or (**f**) mCherry in PBS reveal detectable in-flow signals following a Gaussian-shaped distribution in the associated histograms.

Figure 2a,b shows the background signal level of the aqueous solution used for polystyrene bead and carbon particle measurements (Fig. 2a), as well as a phosphate-buffered saline (PBS) solution used for the *E. coli* measurements (Fig. 2b). In both cases, no signals were detected over 10 s, which allowed a transit-analysis based on thresholds of 400 a.u. for OA and 5 a.u. for LS. The above-threshold OA signals of polystyrene beads (Fig. 2c) have a value of 1.096 × 10^3^ ± 73.1 a.u. and a LS signal of 13.6 ± 1.24 a.u. (mean ± standard error). The signal deviations may result from polystyrene beads floating through the interrogation area at different positions and, thus, experiencing different fluence values of the optical excitation cone. For the current system this signal deviation of about ± 8% defines the maximal achievable precision without signal correction in determining signals from a solution of specimens that are homogenous in size and signal. Analogous measurements of carbon particles (Fig. 2d) indicate an OA signal of 1.806 × 10^3^ ± 190.7 a.u. and a LS signal of 11.1 ± 3.19. The increase in spread of OA and LS signal amplitudes is due to the broad size distribution of carbon particles of 2–12 µm. As an example of an in vitro application we performed similar measurements on mixtures of *E. coli* bacteria expressing either mCherry (UniProtKB: X5DSL3) with an absorption of 72,000 M^−1^ cm^−1^ at 587 nm or mKusabira Orange1 (mKO; UniProtKB: Q6I7B2) with 51,600 M^−1^ cm^−1^ at 548 nm. Both plots in Fig. 2e,f reveal that the system’s sensitivity is high enough to measure single-cell transits and sense both the OA signals from the chromoprotein and LS signals from the cell body.

### OA-FCM measurements of genetically-encoded contrast

Besides the sensitivity of the system, its ability to discriminate different populations in a mixture of cells is crucial, as described in the preprint of this manuscript^18^. Towards this end, we assessed if we could distinguish *E. coli* cells expressing the above-mentioned chromophore proteins mCherry and mKO in a mixture in-flow based on their different absorption values, and hence their OA signals at the excitation wavelength of 532 nm. Considering both the associated absorption values at 532 nm (i.e. mCherry: 40%—> 28,800 M^−1^ cm^−1^; mKO: 58.8%—> 30,340 M^−1^ cm^−1^) as well as their respective quantum yield (i.e. mCherry: 0.22; mKO: 0.6), optoacoustic relevant absorption coefficients can be approximated as 22,000 M^−1^ cm^−1^ for mCherry and 12,000 M^−1^ cm^−1^ for mKO. Hence, mCherry is expected to generate OA signals that are approximately 1.8 times stronger those from mKO, while LS signals from both cultures are expected to resemble each other. We measured pure solutions of mCherry or mkO expressing cells, as well as a 1:1 mixture of the stock solutions. The pure solutions showed above-threshold peaks of mCherry-cells having an OA signal of 837.5 ± 153.6 a.u. and a LS signal of 13.7 ± 4.8 a.u., whereas mKO-cells show an OA signal of 666.5 ± 91.78 a.u. and a LS signal of 12.1 ± 5.1 a.u (see Fig. 2e,f). After subtracting the above-mentioned OA background values of 400 a.u. (used for thresholding), the mean OA signals become 437.5 ± 153.6 a.u. for mCherry and 266.5 ± 91.78 a.u. for mKO, thereby yielding a ratio of ~ 1.65 with a t-test *p* value of 0.39 (N_mkO_: 1396, N_mCherry_: 2108). After subtracting the above-mentioned LS background values 5 a.u (also used for thresholding), the mean LS signals become 8.7 ± 4.8 a.u. for mCherry and 7.1 ± 5.1 a.u. for mKO, thereby approximating a ratio of ~ 1.23 with a t-test *p* value of 0.82 (N_mKO_: 2108, N_mCherry_: 1396). As expected, the LS signal for both stocks resemble one another when an identical cell type is used, whereas the OA signal of cells expressing mCherry is larger than that of cells expressing mKO. The large deviations, especially for the OA signal, might be due to differences in expression levels of the chromoproteins within the cellular population. However, neither individual modality achieved statistical significance in separating mKO from mCherry, which renders the need for a dual-modal reading to yield accurate separation.

**Figure 3:**
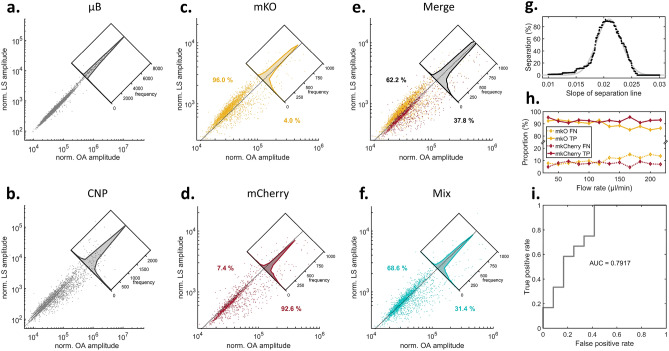
Signal distribution of in-flow single transit events of *E. coli* cells expressing optoacoustic labels enabled separation by applying linear discrimination. (**a**) Norm. OA signals against norm. LS signals of µB and (**b**) CNP, and (**c**) *E. coli* cells expressing mKO. Inset depicts separation by linear discrimination of 96.0%. (**d**) Analogues analysis of *E. coli* cells expressing mCherry yield a discrimination of 92.6%. (**e**) Data merge of the separate measurements and (**f**) measurement of cell stock mixture (mixing ratio 1:1) of mKO and mCherry *E. coli* cells yield a linear discrimination of ~ 2:1 resembling the ~ 3:1 concentration difference in stock solutions. (**g**) Varying the slope of the linear discrimination and Gaussian fitting peaked in ~ 0.0209 a.u. with an average separation of ~ 92.5%. (**h**) Flow speed analysis shows consistent linear discrimination by constant true-positive (TP) and false-negative (FN) assignments of signals. (**i**) ROC analysis reveals an AUC of ~ 0.8.

Figure 3a–d includes merged data of three 1 min-measurements per flow rate for cells and two 1 min-measurements per flow rate for µB and CNP, carried out at 12 different flow speeds ranging from ~ 33 to ~ 214.5 µL/min. A sharp linear relationship between LS and OA signals can be observed (Fig. 3a) when referencing each OA transit signal of 10 µm blacked µBs to the associated LS signal, which supports the validity of correcting OA-signals by LS encoding for the object size and relative position in the interrogation area. Linear regression yielded a ratio of 0.00978 between LS and OA. An analogous analysis of 2–12 µm CNPs (Fig. 3b) affords a broader spread of the signals, which indicates a more inhomogeneous distribution of size and shape when compared to the µB measurement. Linear regression yielded a ratio of 0.01248 between LS and OA. Figure 3c,d shows the scatter plots of *E. coli* cells expressing mKO (Fig. 3c) or mCherry (Fig. 3d), respectively. Linear regression yielded a ratio of 0.0242 for mKO and 0.0186 for mCherry. When compared to µB and CNP, we found that *E. coli* cells not only generated weaker LS and OA signals in general, but also weaker OA signals relative to the associated LS signals. The insets in Fig. 3c–f depict a signal histogram based on an inclined axis, which also enables linear discrimination with a separation precision of > 92% for both cases. Furthermore, we merged the two data sets of mKO and mCherry and found a partitioning at ~ 2:1 (62.2% vs. 37.8%) via linear discrimination (Fig. 3e), similar to what was measured in a 1:1 mixture of the cell stock solutions (68.6% vs. 31.4%, Fig. 3f). The found partitioning resembles roughly the predetermined cell concentration ratios of the stock solutions, which are ~ 3:1 (7.4 × 10^8^ cfu/mL for mKO and 2.6 × 10^8^ cfu/mL); deviations might be introduced by unequally diluting the stock solutions before measurements. For system optimization, we combined all recorded data and tried to vary the slope of linear discrimination via fitting a Gaussian curve (Fig. 3g), which was found best for 0.0209 a.u. and led to an overall discrimination of ~ 92.5%. The accuracy of the separation slope was tested by calculating an optimal separation slope for all set of measurements (3 merged data sets at each flow speed) individually. This calculation yielded a standard deviation of 0.0003757 a.u. (exemplary 10 s-trajectories of the measurements shown in the Supplementary Information). A separation accuracy of > 80% true-positives (TP) and < 20% false-negatives (NG) was consistently achieved at different flow speeds ranging from ~ 33 to ~ 233 µL/min (Fig. 3h). We achieved a separation reliability of ~ 0.8 by area-under-curve (AUC) of a true- vs. false-positive comparison (receiver operator characteristics analysis (ROC), Fig. 3i).

## Discussion

In this work, we presented a pilot study of an OA-FCM for the in vitro screening of OA contrast, in single cells and in-flow. Our OA-FCM allows precise characterization of the OA contrast of cells by referencing in-flow OA signals to simultaneous LS to account for size, position and excitation light flux. Our OA-FCM demonstrates that OA contrast can be accurately determined for single-cells in-flow, paving the way for the development of high-throughput OA cell sorters for directed evolution of transgene reporters tailored for OA imaging.

We showed that the sensitivity of both modalities enabled the detection of objects as small as single cells transiting through the system’s interrogation area in a microfluidic arrangement. Our OA-FCM prototype uses 45° LS as an independent modality that increases the reliability of measuring OA signals of single cells in high-throughput flow setups. It therefore overcomes previous attempts of optoacoustic flow cytometers based on fluorescence- or ultrasound-referencing and achieves for the first-time precise discrimination of intracellular OA contrast of genetically-encoded proteins.

Our OA-FCM has a significantly smaller optical interrogation area compared to the channel cross section (~ 2%), which agrees with the estimated fraction of detected microbeads (2.36%) in our test solution (Fig. 2c, Test solution: ~ 4.55 × 10^7^ particles/mL; 100 dilution, 150 µL/min; 10 s time window; expected number of signals: 11,375; average of measured signals: 268 signals). In the future, the flow stream containing cells or particles could be reduced in size by using either smaller microfluidic channels or microfluidic focusing via sheath flow arrangement to enhance the detected fraction.

For more comprehensive assessments and quantification of a reporter’s suitability for OA imaging, other signals could be read alongside OA and LS. For example, simultaneously detecting fluorescence emitted by the absorbing entity would enable further exploration of optical characteristics (e.g. quantum yield, life time, bleaching). Regarding the intracellular OA readings, the simultaneous recording of additional wavelength could allow appropriate estimation of the cell's condition in respect to its expression level and molecular composition or adaption of the analysis to different cell types. Additionally, recording LS signals at more angles, i.e. 45° and 135°, would facilitate a more precise deduction of the objects size. Further, full automation of signal analyses and cell-sorting would allow follow-up models to adapt concepts such as directed-evolution for protein-engineering of novel transgene contrast agents.

Precise in-flow readouts could also be useful for applications such as the characterization of cells by their absorption fingerprint, inferred from the OA signals at specific wavelengths. OA-FCMs could potentially exploit the absorption-based contrast of OA for label-free sensing of a cell’s contents, such as lipids, proteins, and carbohydrates^19^, enabling sorting based on the distinct absorption characteristics of individual cells^20^. This can be used to read out for instance, the cells energy metabolism (oxidative phosphorylation, FAD, NAD and *Cyt c*) or infer erythrocyte related diseases, such as, hyperlipidemina, plasmodium infections, or hemoglobin defects. High-throughput, direct characterization of biomolecules is extremely challenging, as they do not typically autofluoresce or absorb in the range of standard excitation sources used for OA and fluorescent methods (e.g. 500–1000 nm). Future OA-FCM prototypes could integrate freely tunable optical excitation to shift the applied spectral range towards the more applicable near- and mid-infrared ranges and capitalize on approaches for simultaneous wavelength multiplexing.

The herein presented OA-FCM is an essential step towards enabling the high-throughput screening of cells based on their OA signal, potentially fostering the development of novel transgene labels tailored for OA imaging. OA-FCM has a strong potential to not only impact the optoacoustic field, but also a wide range of applications for life sciences and medical practice by revealing cellular information.

## Methods

As schematically depicted in Fig. 1a and described in the preprint of this manuscript^18^, the OA flow cytometer (OA-FCM) utilizes the optical excitation of an actively triggered 532 nm laser (SPOT-10-200-532, Elforlight Ltd; pulse length: 1.2 ns; pulse energy at the sample: 8.7 nJ, repetition rate: 50 kHz), which is spatially filtered by a 25 µm pinhole, enlarged by a telescopic arrangement of plan-convex lenses, guided into an inverted microscope (Axio Observer, Zeiss), and focused by a microscopic objective (10×, 0.45 NA, Zeiss) to a diffraction limited spot. By that, the optical excitation is focused into a microfluidic chip (01-0175-0138-02, microfluidic ChipShop, Jena, Germany) with a flow channel of 1000 µm width and 100 µm height. The channel bottom, through which the optical excitation is focused, consists of a 1 mm thick cyclo-olefine co-polymer, while the top side, facing both sensing detectors is made of the same polymer but with only 140 µm thickness. For OA sensing, we equipped a high-frequency ultrasound transducer (HFM23, SONAXIS, France; focal length: 3 mm, bandwidth: 10–110 MHz) and for LS sensing an optical fiber (M15L01, Thorlabs) tilted into an 45° angle with respect to the optical axis connected to a high-speed photodiode (DET10A, Thorlabs) equipped with a shortpass optical filter to spectrally block potential fluorescence light. The facet of the fiber was immersed into the water droplet for acoustic coupling to ensure equalized recording of LS signals. The microfluidic chip is connected to a syringe pump (NE-1000, New Era Pump Systems Inc, USA) in order to push the suspensions with a defined flow speed through the channel. Upon a trigger signal sent out by a function generator (DG1022, Rigol) to the laser as well as the recording DAQ (ADQ412, SP Devices; sampling speed: 450 MS/s; dynamic range: ± 750 mV; 2 channels; bit depth: 12 bit), both the amplified (AU-1291, Miteq; 63 dB) OA as well as the LS signal are recorded in a high-speed streaming like process based on temporary buffers only limited by the computer memory (i.e. 64 GB) leading to a data transfer of ~ 100 MB/s. To avoid recording ultrasound reflections occurring at the walls of the microfluidic channel or within the polymer lid facing towards the transducer, the recording window was adjusted to collect only signals directly approaching the transducer. The OA signals are further bandpass filtered in the range of 5–90 MHz. Both signals are then projected using their maximum amplitude of dedicated time windows covering the corresponding signals. The control as well as the subsequent analysis of the yielded flow cytometry trajectories is carried out in Matlab^18,21–24^.

For characterizing and calibrating the OA-FCM system and as described in the preprint of this manuscript^18^, we first recorded flowmetry trajectories of suspensions of 10 μm blacked microbeads (μB) and 2–12 μm carbon-nano-particles (CNPs). In all cases, the suspensions were diluted and replenished with ~ 1%vol. TWEEN™ 20 (Sigma-Aldrich, Germany) to reduce the surface tension for preventing agglomeration, and with the appropriate amount of sodium-polytungstate (Sigma-Aldrich) for increasing the density of the medium to prevent sedimentation. The required concentration of sodium-polytungstate was determined via calculating the volume fraction of the particles. The prepared suspensions were rested for 1 h to verify proper adjustment of the density. For cell measurements, coding sequences for mKO (UniProtKB: Q6I7B2) and mCherry (UniProtKB: X5DSL3) were synthesized codon optimized for E. coli (GeneArt, ThermoFisher) and inserted in pET21a(+) vector (Novagene) using the NdeI and XhoI (ThermoFisher) restriction sites. Proteins were expressed in BL21 DE3 (New England Biolabs) at 37 °C and 180 rpm after Isopropyl-thiogalactoside (IPTG) induction. Cell density and protein expression was monitored by optical absorption measurements at 600 nm and fluorescence measurements at the respective peak absorption wavelengths of the two proteins^18^. All flow measurements had a duration of 1 min.

## Supplementary Information

Supplementary Information

## Data Availability

The datasets of this study are available from the corresponding author on reasonable request.

## Abbreviations

AUC: Area under curve
CNP: Carbon nano particles
*E. coli*: *Escherichia coli*
FN: False negative
LS: Light scattering
µB: Microbeads
mKO: mKusabira Orange1
OA: Optoacoustic
OA-FCM: Optoacoustic flow cytometer
ROC: Receiver operator characteristics
TP: True positive
