# Supplementary material for: In vitro optoacoustic flow cytometry with light scattering referencing
Source: Sci Rep. 2021 Jan 26;11:2181. doi: 10.1038/s41598-021-81584-y (PMC7838204; doi:10.1038/s41598-021-81584-y)

**SUPPLEMENTARY INFORMATION**

**to**

**In vitro optoacoustic flow-cytometry with light scattering referencing**

*
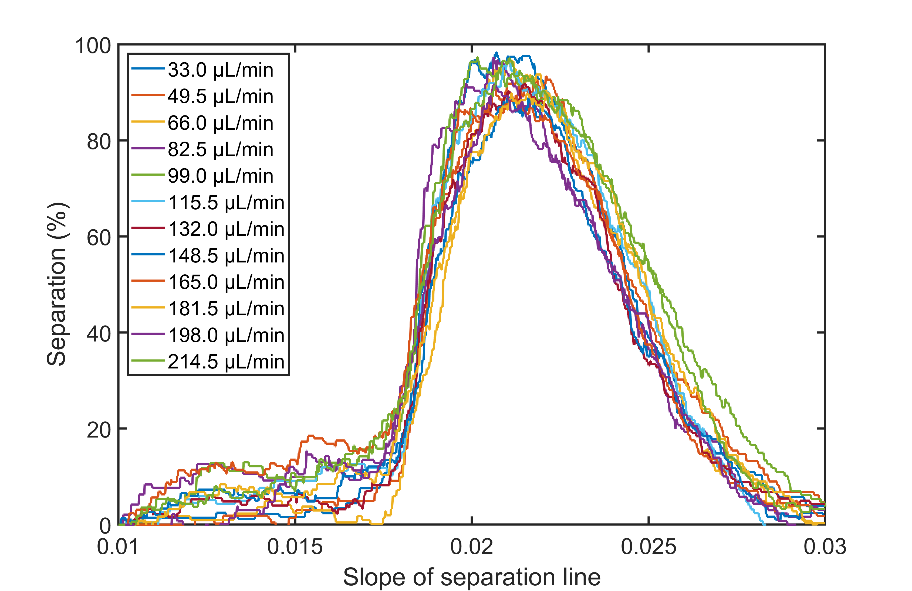
*Markus Seeger, Andre C. Stiel and Vasilis Ntziachristos

***Supplementary Fig. 1 Separation slope at different flow rates.*** *The slope for separating mkO and mCherry was individually optimized for 3 measurements per flow rate at 12 different flow rates whith each measurement lasting 1 min. In all cases, a separation of ~90% was achieved.*

***Supplementary Tab. 1 Separation slope at different flow rates.*** *An optimum separation slope was generated for each of the 12 data sets individually. When combining all data sets, a total mean value 0.0209 with a standard deviation of 0.0003757 was achieved.*

| **Flow rate** | **Slope** | **R** |
| --- | --- | --- |
| 33.0 µL/min | 0.02059 | 0.965 |
| 49.5 µL/min | 0.02082 | 0.962 |
| 66.0 µL/min | 0.02093 | 0.964 |
| 82.5 µL/min | 0.02127 | 0.936 |
| 99.0 µL/min | 0.02081 | 0.945 |
| 115.5 µL/min | 0.02171 | 0.967 |
| 132.0 µL/min | 0.02062 | 0.970 |
| 148.5 µL/min | 0.02067 | 0.971 |
| 165.0 µL/min | 0.02142 | 0.955 |
| 181.5 µL/min | 0.02098 | 0.980 |
| 198.0 µL/min | 0.02152 | 0.975 |
| 214.5 µL/min | 0.02081 | 0.978 |

***Supplementary Fig. 2 Exemplary 10s-trajectories of E.coli cells expressing mkO at flow rates between 33.0 and 214.5 µL/min.***


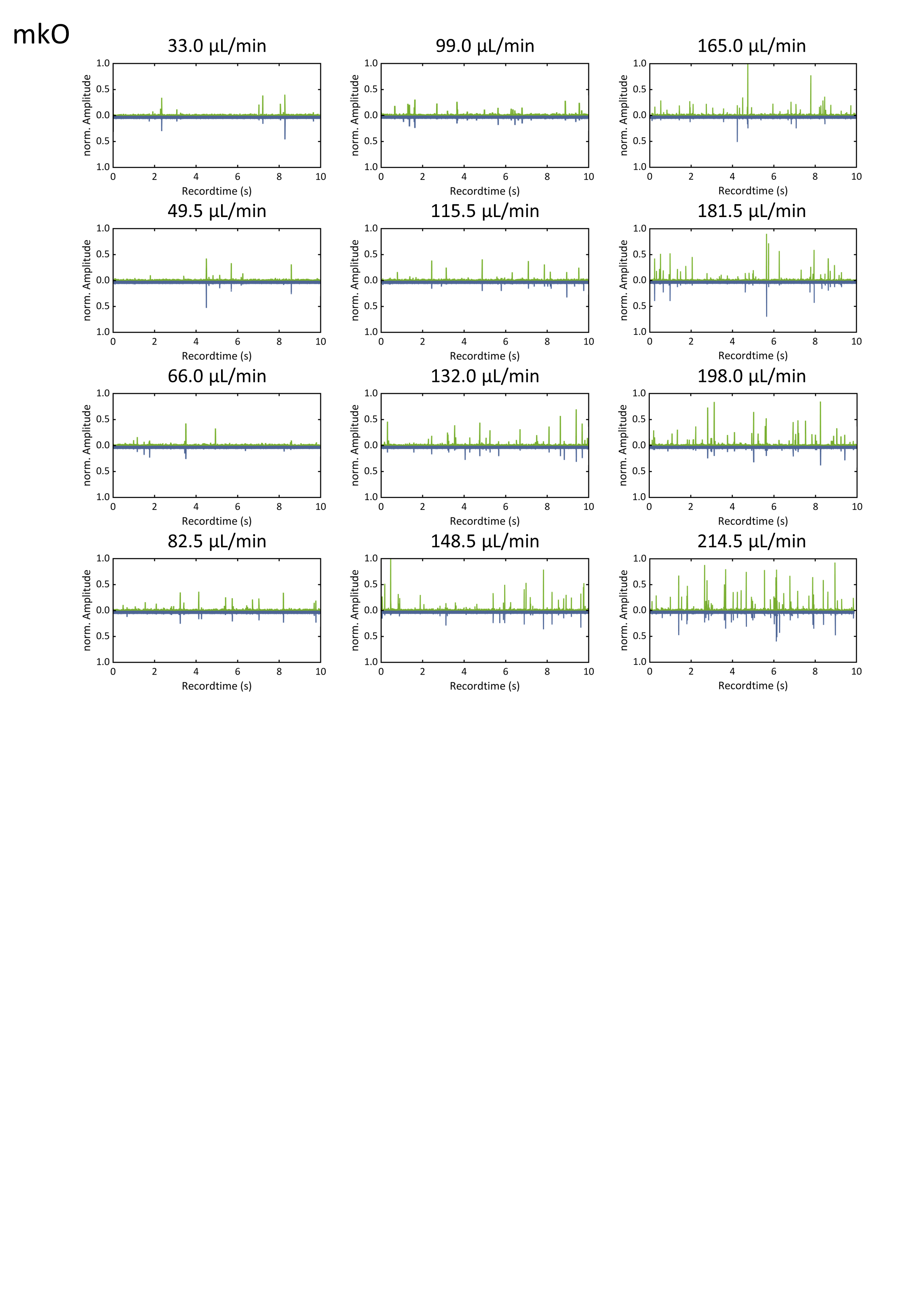


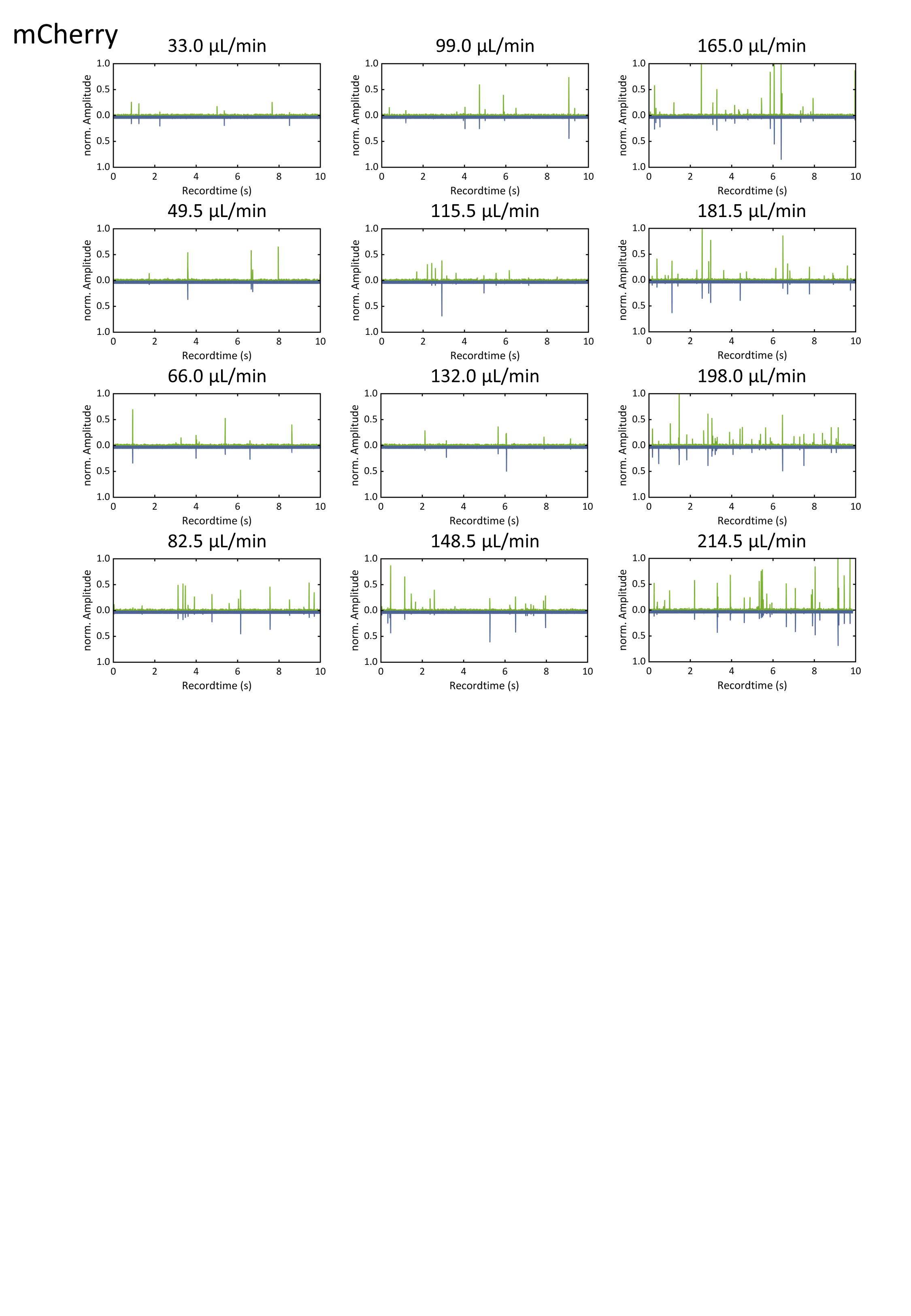


***Supplementary Fig. 2 Exemplary 10s-trajectories of E.coli cells expressing mCherry at flow rates between 33.0 and 214.5 µL/min.***

***Supplementary Fig. 2 Exemplary 10s-trajectories of a mixture of E.coli cells expressing mkO or mCherry at flow rates between 33.0 and 214.5 µL/min.***


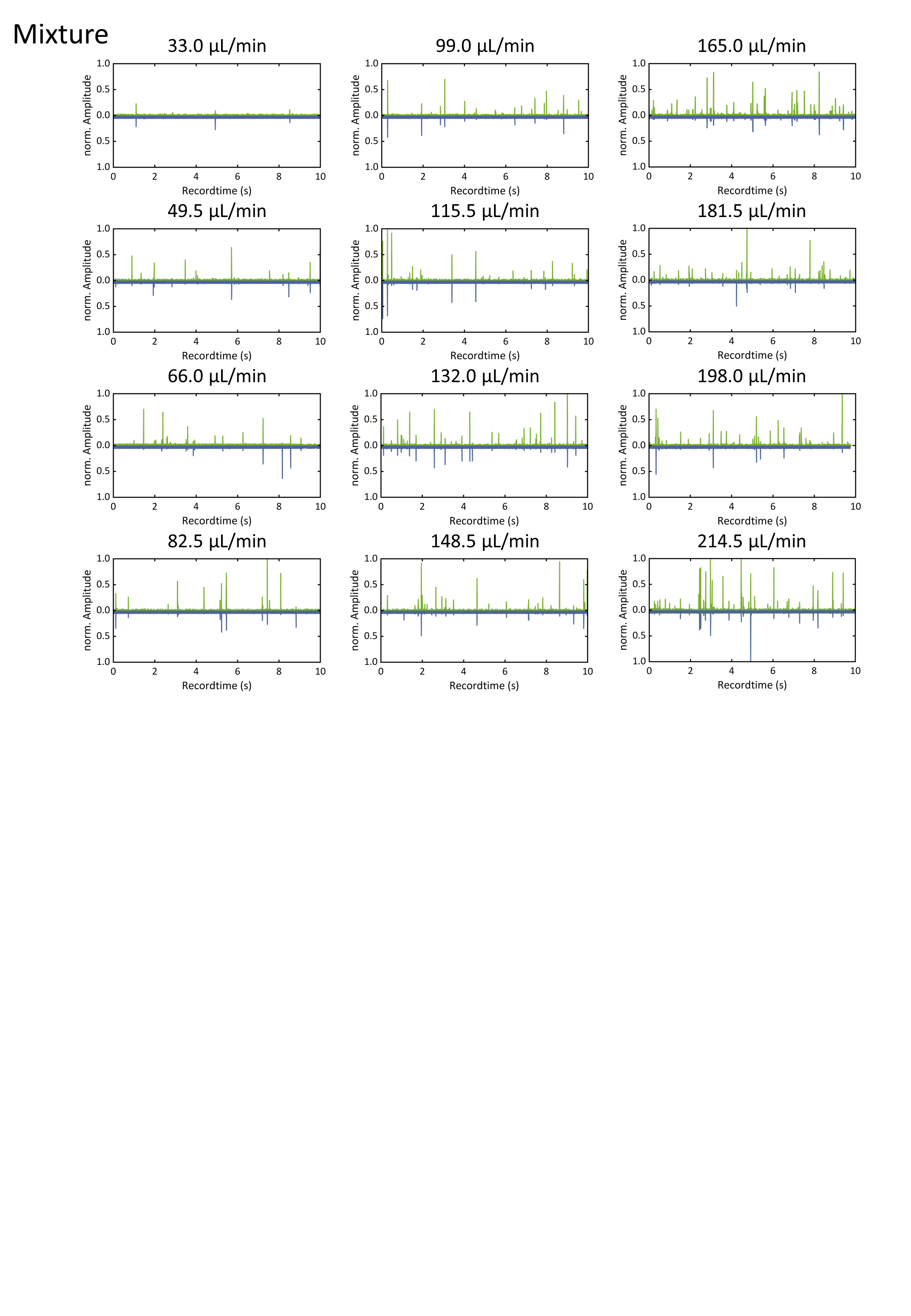

Supplement: Supplementary file 1 — Supplementary Information [file 41598_2021_81584_MOESM1_ESM.docx]
